# Pulmonary Artery Banding in a Cat with Atrioventricular Canal Defect Type A with Concurrent Muscular Septal Defect

**DOI:** 10.3390/ani15071044

**Published:** 2025-04-04

**Authors:** Olga Szaluś-Jordanow, Krzysztof Zdeb, Wojciech Mądry, Michał Buczyński, Anna Świerk, Zofia Nowek, Agata Moroz-Fik, Michał Czopowicz

**Affiliations:** 1 Department of Small Animal Diseases with Clinic, Institute of Veterinary Medicine, Warsaw University of Life Sciences-SGGW, Nowoursynowska Str. 159c, 02-776 Warsaw, Poland; 2 Anicura Legwet Clinic, Piotra Wysockiego Str. 31, 05-120 Legionowo, Poland; krzysztof.zdeb@legwet.pl (K.Z.); kornatowska.an@gmail.com (A.Ś.); 3 Department of Heart, Chest and Transplant Surgery, Medical University of Warsaw, Żwirki i Wigury 63A, 02-091 Warsaw, Poland; madwoj1@onet.eu (W.M.); mbuczynski2@wum.edu.pl (M.B.); 4 Division of Veterinary Epidemiology and Economics, Institute of Veterinary Medicine, Warsaw University of Life Sciences-SGGW, Nowoursynowska Str. 159c, 02-776 Warsaw, Poland; zofia_nowek@sggw.edu.pl (Z.N.); agata_moroz@sggw.edu.pl (A.M.-F.); michal_czopowicz@sggw.edu.pl (M.C.)

**Keywords:** atrioventricular septal defect, AVSD, pulmonary artery banding, PAB, cat

## Abstract

Background: This case report describes the surgical management of pulmonary artery banding (PAB) in a cat diagnosed with an incomplete atrioventricular canal (AVC) defect and a concurrent muscular septal defect. It highlights the use of PAB as a palliative treatment to manage this rare congenital heart condition in companion animals. Case Presentation: A 9-month-old European long-haired male cat presented with clinical signs of heart failure. Pharmacological treatment with sildenafil and furosemide failed to stabilize the patient’s condition. Echocardiographic assessment revealed an incomplete AVC type A and a muscular septal defect. Pulmonary artery banding was performed to reduce pulmonary blood flow and alleviate heart failure symptoms. Pre- and postoperative echocardiographic evaluations were conducted to monitor structural and functional changes in the heart. Post-surgical outcomes included a marked reduction in the size of the right atrium and ventricle and a stable pulmonary artery flow velocity of 3.8 m/s. The cat has remained in very good condition without the need for pharmacotherapy for 13 months post-surgery and is still alive at the time of reporting. Conclusions: PAB proved effective as a palliative intervention for managing an incomplete AVC in this feline patient. The successful outcome suggests that PAB may offer significant long-term benefits and improved survival in selected cases of incomplete AVCs in cats.

## Simple Summary

A young, long-haired cat was diagnosed with a complex congenital heart defect, causing difficulty in breathing and fluid accumulation in the abdomen. Despite medical treatment, the cat’s condition worsened, leading to severe heart failure. A surgical procedure called pulmonary artery banding was performed to reduce excessive blood flow to the lungs and relieve strain on the heart. During the operation, a band was placed around the pulmonary artery to control blood pressure and balance circulation between the heart chambers. Following the surgery, the cat showed significant improvement. Its breathing normalized, fluid buildup resolved, and no further medication was required. The cat remained in excellent health for twelve months after surgery, able to run and jump without difficulty. This case highlights the potential of pulmonary artery banding as a valuable surgical option for managing congenital heart defects in cats, offering them a better quality of life and improved long-term survival.

## 1. Introduction

The atrioventricular septal defect (AVSD) is a group of complex congenital heart defects resulting from the abnormal development of endocardial cushions. These structures arise due to the expansion of gelatinous substances during embryogenesis and later develop into material forming atrioventricular (AV) valves, semilunar valves, and the heart septum. The dorsal endocardial cushion connects with the primary septum to close the primary opening in the interatrial septum. The dorsal and ventral cushions fuse to divide the common atrioventricular canal (AVC) into two separate channels: left and right [1]. Studies in mice have shown that disruptions in the development of mesenchyme tissue, which is part of the development process and corresponds to the interatrial septum complex, may be the cause of this heart defect [2,3]. The concept of the AVC encompasses a group of defects in which a single common AV connection replaces two separate AV connections that are normally present. Depending on the severity of the changes, the AVC can be classified as complete, transitional, or incomplete. The complete AVC includes a defect in the primary interatrial septum (primum ostium), a defect in the interventricular septum (IVS) (high, just below the AV valves), and abnormal development of the AV valves, typically consisting of a single valve with a five-leaflet valve, located above both ventricles, guarding the common AV orifice [4,5]. Leaflets close to the IVS are called “bridging leaflets”, as they are often overly in the septal plane. In dogs and cats, the occurrence of an incomplete canal has been documented, which includes a large defect in the primum interatrial septum and abnormalities in the structure of the mitral valve—the anterior leaflet of the mitral valve is divided into two, resulting in the presence of three leaflets of the mitral valve—usually called the “cleft mitral valve” [3,6]. The transitional canal is a form between a complete and partial canal. Usually, a significant deficit of the IVS tissue exists, but interventricular communication is partially or completely closed by septal attachments of the so-called bridging leaflets—leaflets of the common AV valve located close to the IVS (the presented case) [7]. In the complete AVC, the mitral and tricuspid valves are located at the same level [8], while in the transitional AVC, the mitral valve is displaced towards the apex of the heart. A case of an incomplete AVC in a dog with a defect in the IVS has been described [1]. In cats, AVSDs account for 5–10% of all congenital heart defects [9]. A complete AVC can also be divided into three types: A, B, and C, according to Rastelli (1966) [10], depending on the structure of the common AV valve. In type A, its anterior leaflet attaches to the crest of the IVS through a thickened chordae tendineae. In type B, the anterior leaflet is anchored to or near the part of the IVS belonging to the right ventricle through the papillary muscle. In type C, it has no connection with the IVS and freely floats inside the heart cavity [9]. In cats, isolated cases of an AVC have been described with the coexistence of a triatrial heart [6]. Clinical symptoms depend on the severity of the defect. In most animals, an AVC is diagnosed in the first year of life. An AVC leads to mixing oxygenated and deoxygenated blood within the heart. As long as the pressure in the left ventricle is higher than in the right ventricle, the left-to-right shunt results in cardiorespiratory failure (due to pulmonary hyper perfusion and ventricular volume overload). Gradual destruction of pulmonary vasculature results in pulmonary hypertension (PH). When the pressure in the right ventricle exceeds the pressure in the left ventricle, the shunt reverts into right-to-left and non-oxygenated blood is directed to the systemic circulation. This leads to Eisenmenger syndrome, with cyanosis easily detectable in a clinical examination. The anatomical abnormalities of the AV valve(s) cause the AV dysfunction, mainly incompetence, and exacerbate heart failure as well as accelerate the development of PH [11]. Other clinical symptoms include decreased exercise tolerance, dyspnea, ascites, cough, emaciation, lethargy, syncope, and increased resting respiratory rate [3,7,11]. Syncope is most commonly a consequence of PH [12]. During auscultation, a heart murmur may be present along with crackles upon auscultation of the lung fields [3], although the authors have observed patients without heart murmurs as in the presented case. The primary method for detecting an AVC is transthoracic echocardiography [1,9,10]. The occurrence of this defect has also been reported in animal species other than companion animals [13,14,15].

In human medicine, this heart defect is commonly corrected through surgery. However, while similar procedures could technically be performed on animals, they are rarely conducted due to the high costs involved. Pharmacological management mainly includes the treatment of heart failure: diuretics, antiarrhythmic drugs, and sildenafil [3,16,17].

In companion animals, an AVS constitutes an extremely rarely diagnosed congenital heart defect. Based on the main author’s experience over the past 10 years, out of 19,200 echocardiographically examined patients, this defect was confirmed in only three cats and not in any dog. This is most likely due to the fact that animals with such severe defects do not survive long enough to undergo echocardiographic examination, and these defects may be lethal. Despite the rarity of an AVS, there is a need to explore potential treatment options to improve survival and quality of life in affected patients.

Therefore, this study aims to evaluate the feasibility and effectiveness of pulmonary trunk banding as a surgical intervention for AVSs in cats. This procedure aims to redirect blood flow to balance blood volume between the pulmonary and systemic circulation, potentially offering a life-extending option for cats diagnosed with this severe congenital defect.

## 2. Case Presentation

**Figure 1:**
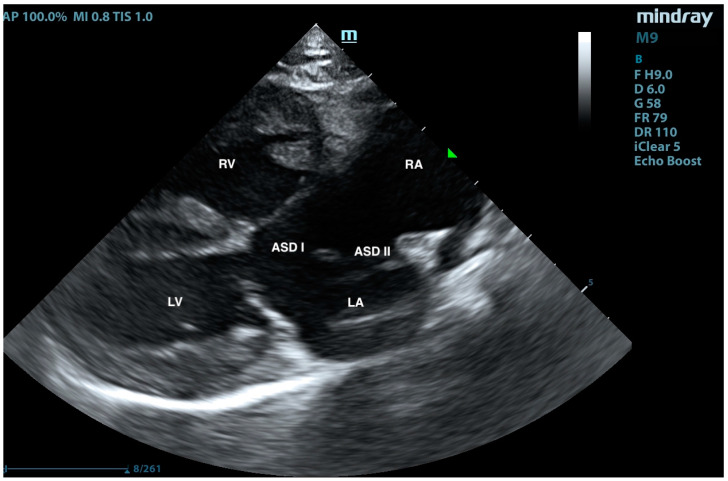
Right-sided long-axis view, diastolic phase. RA—right atrium, LA—left atrium, RV—right ventricle, and LV—left ventricle. The closed atrioventricular valve is visible, along with part of the interatrial septum showing ASD I—atrial septal defect I—and ASD II—atrial septal defect II. RV and RA enlargement is visible.

**Figure 2:**
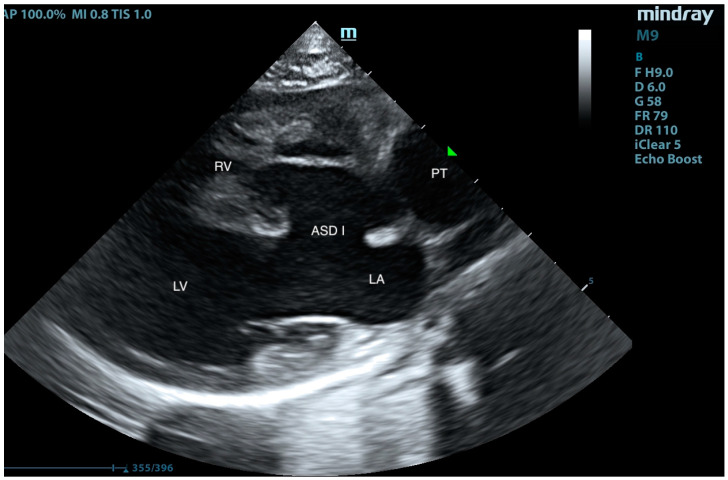
Right-sided long-axis view, diastolic phase. LA—left atrium, LV—left ventricle, RV—right ventricle, PT—pulmonary trunk, and ASD I—atrial septal defect I. The open left atrioventricular valve is visible.

**Figure 3:**
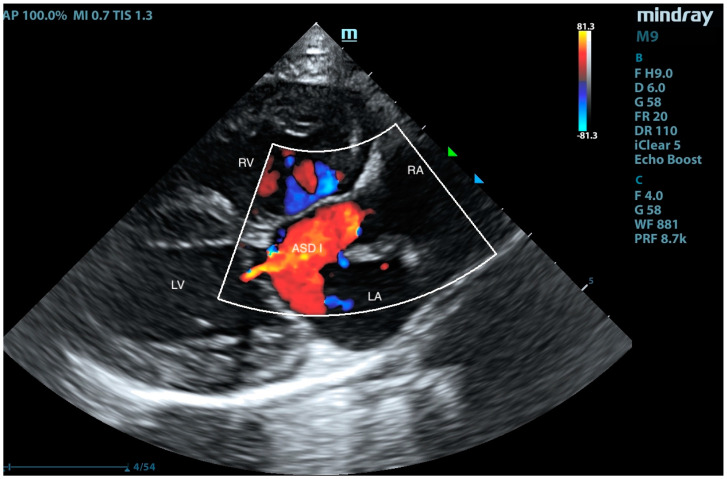
Right-sided long-axis view. RA—right atrium, LA—left atrium, RV—right ventricle, and LV—left ventricle, systolic phase. Left-to-right blood flow is visible in color Doppler through ASD I.

**Figure 4:**
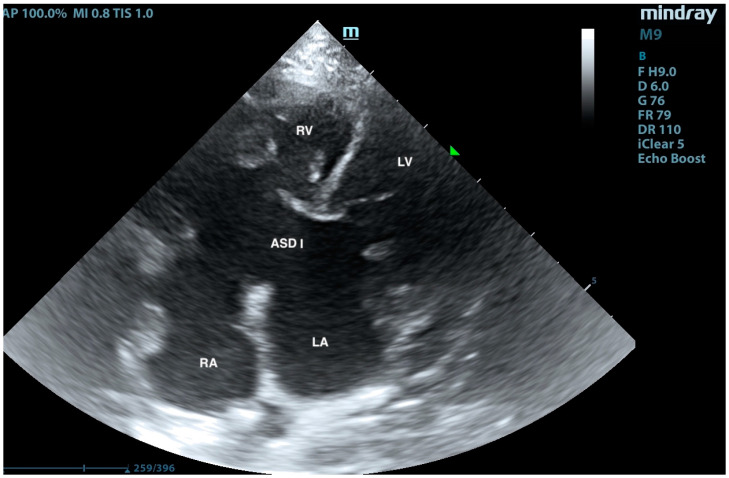
Left-sided four-chamber view. LA—left atrium, RA—right atrium, RV—right ventricle, LV—left ventricle, and ASD I—atrial septal defect I.

**Figure 5:**
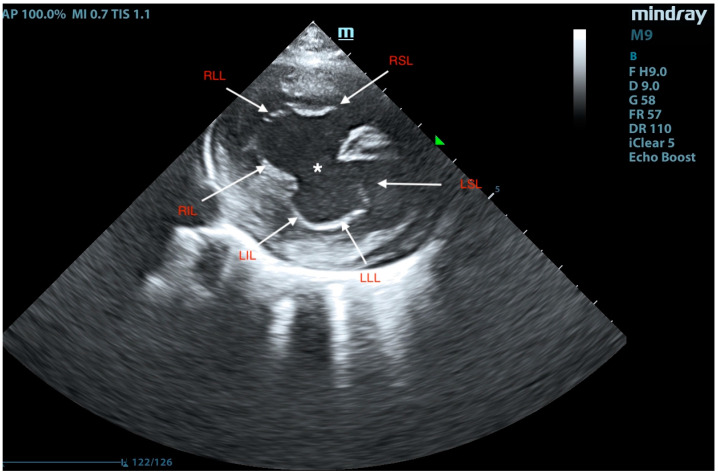
Right-sided short-axis view. The open common atrioventricular valve is visible, with RIL—right inferior leaflet, LIL—left inferior leaflet, LLL—left lateral leaflet, LSL—left superior leaflet, RSL—right superior leaflet, RLL—right lateral leaflet (commissures), and an asterisk marking the interatrial septal defect.

**Figure 6:**
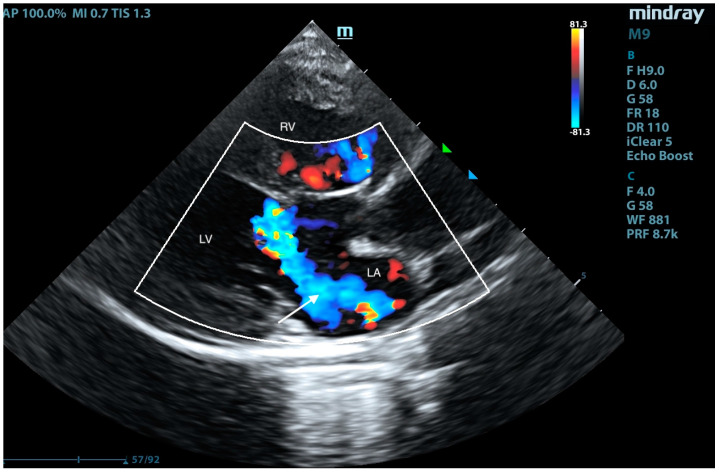
Right-sided long-axis view. LA—left atrium, RV—right ventricle, and LV—left ventricle, systolic phase. A regurgitant jet into the left atrium is visible despite the closed left atrioventricular valve (arrow). No clear VSD shunt is visible at the junction of the bridging leaflets with the IVS crest.

**Figure 7:**
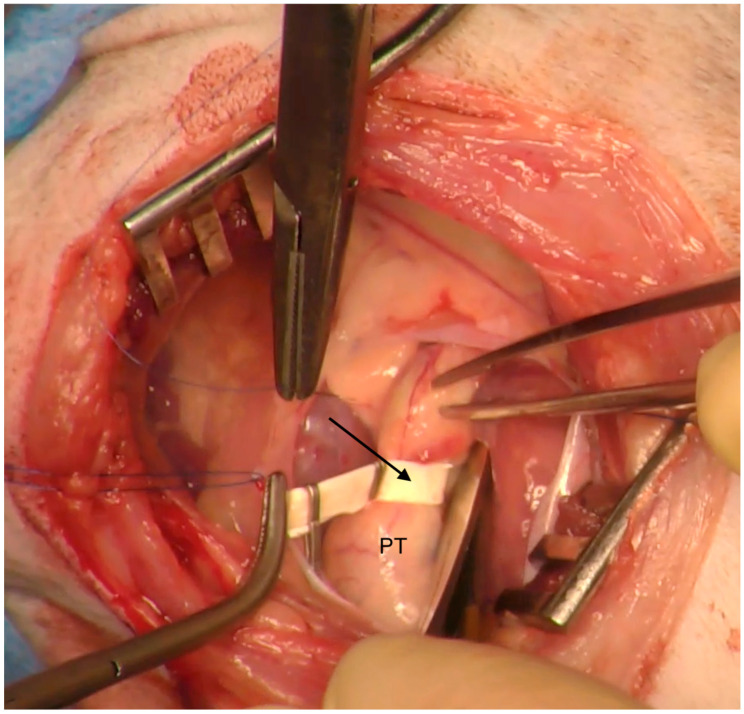
Intraoperative photograph: PT—pulmonary trunk; arrow indicates the Gore-Tex band placed to reduce the lumen of the pulmonary trunk.

**Figure 8:**
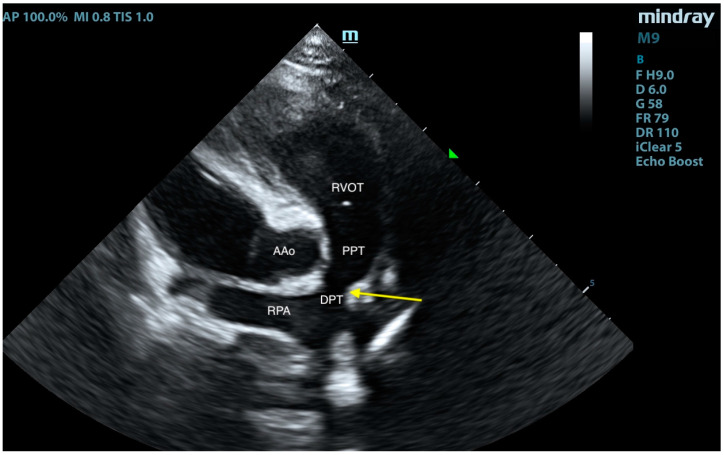
Right-sided short-axis view at the level of the pulmonary trunk. RVOT—right ventricular outflow tract, PPT—proximal pulmonary trunk, DPT—distal pulmonary trunk, AAo—ascending aorta, and RPA—right pulmonary artery. The arrow points to the site of surgical pulmonary trunk banding.

**Figure 9:**
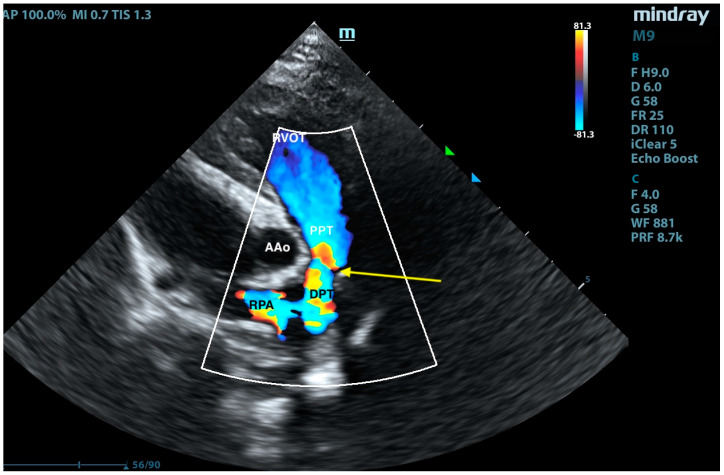
Right-sided short-axis view at the level of the pulmonary trunk with color Doppler. RVOT—right ventricular outflow tract, PPT—proximal pulmonary trunk, DPT—distal pulmonary trunk, AAo—ascending aorta, and RPA—right pulmonary artery. The arrow points to the site of surgical pulmonary trunk banding.

**Figure 10:**
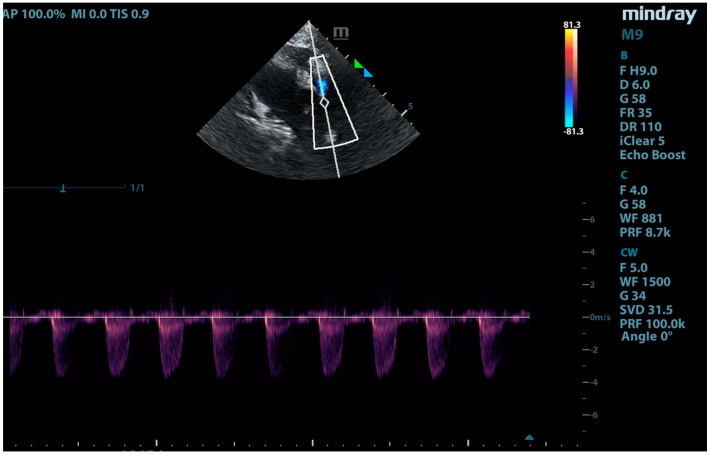
Continuous Doppler imaging showing flow through the banding. Flow velocity of 3.8 m/s.

**Figure 11:**
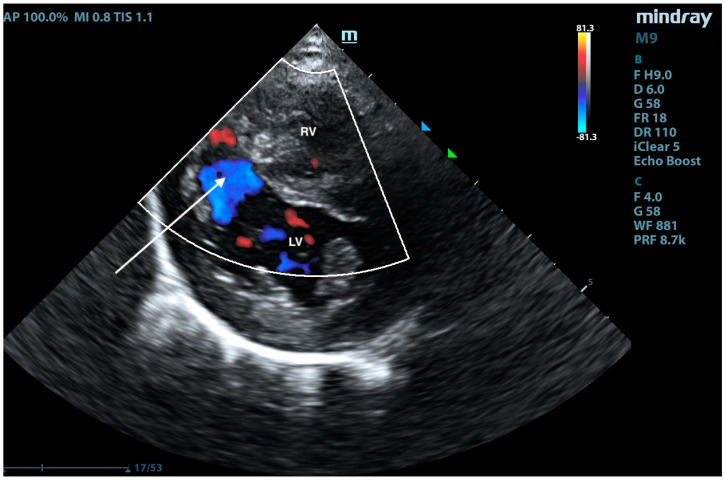
Right-sided short-axis view at the level of the papillary muscles. A right-to-left shunt is visible in color Doppler (arrow). RV—right ventricle; LV—left ventricle.

**Figure 12:**
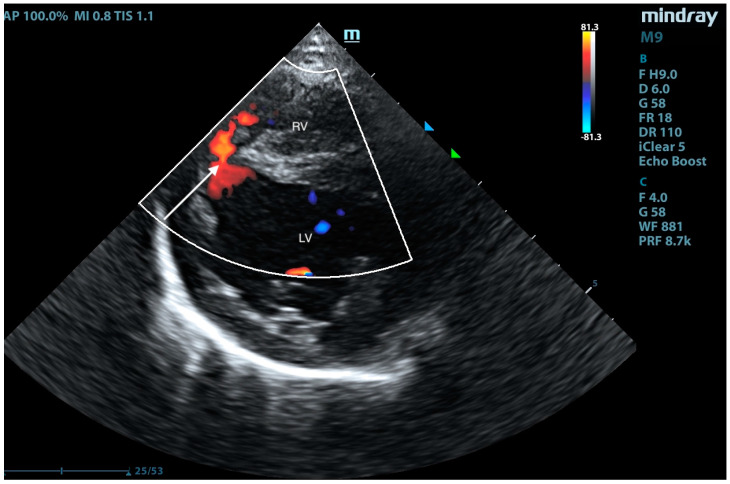
Right-sided short-axis view at the level of the papillary muscles. A left-to-right shunt with low velocity through a large, muscular VSD is visible in color Doppler (arrow). RV—right ventricle; LV—left ventricle.

A European male, long-hair cat, approximately 9 months old, was brought to the veterinary clinic. Since adoption, the cat exhibited rapid breathing, at an average rate of 45/min at rest and up to 70/min after exertion. The patient had previously undergone an echocardiographic examination, revealing an interventricular septal defect. Treatment with 2 mg/kg of sildenafil (Viagra, Pfizer, Sandwich, UK) twice daily and 2 mg/kg of furosemide (Furosoral, Le Vet. Beheer B.V., Oudewater, The Netherlands) twice daily was initiated. On clinical examination, tachypnoea was noted, but no heart murmurs were detected. Other parameters were within reference intervals. Transthoracic echocardiography revealed an atrial septal defect (ASD) of type I and type II (Figure 1, Figure 2, Figure 3, Figure 4 and Figure 5), interventricular septal defect filled with thick, short tendinous cords, and a common atrioventricular valve with regurgitation (Figure 6). Significant enlargement of the right ventricle and right atrium was observed. A diagnosis of AVC defect type A—transitional form, with equal valve distribution between both ventricles, was made. An additional muscular defect in the IVS was visualized. The right-to-left shunt velocity through the ventricular septal defect (VSD) was 0.8 m/s, as visualized by color flow mapping (CFM), indicating equalized pressures in both ventricles, which suggests PH. Sildenafil dose was increased to 3 mg/kg twice daily, and furosemide was changed to torasemide (Upcard, Vétoquinol SA, Paris, France) at 0.3 mg/kg once daily due to difficulties in drug administration. Despite pharmacotherapy, the patient’s condition deteriorated—tachypnoea intensified and ascites developed. The decision was made to proceed with surgical pulmonary artery banding (PAB). On the day of surgery, the patient was in critical condition, with dyspnea and ascites. Before surgery, approximately 500 mL of hemorrhagic fluid was evacuated through abdominocentesis. Furosemide (Furosemidum, Polpharma, Starogard Gdański, Poland) at 4 mg/kg was administered intravenously; oxygen therapy was initiated in an oxygen chamber with a fraction of inspired oxygen (FiO_2_) of 70%. After stabilizing the patient’s condition, surgery was performed. The anesthetic protocol included fentanyl (Fentadon, Dechra, Shrewsbury, UK) at 3 µg/kg IV in a single bolus, midazolam (Dormazolam, Dechra, Shrewsbury, UK) at 0.2 mg/kg IV, alfaxalone (Alfaxan Multidose, Zoetis, Parsippany, New York, USA) at 1.5 mg/kg IV for induction, propofol (Propomitor, Orion Pharma, Warsaw, Poland) at 5 mg/kg IV for induction, and isoflurane (Isoflurin, Geulincx, Tanowo, Poland) for maintenance anesthesia. The percentage of isoflurane vaporized in oxygen during the maintenance of anesthesia ranged from 1.0% to 2.0%, and local anesthesia was used with a serratus plane block using bupivacaine (Bupivacainum Hydrochloricum WZF 0.5%, Polpharma, Starogard Gdański, Poland) at 1.75 mg/kg under ultrasound guidance. A surgical approach was performed in the fifth left intercostal space. After retracting the left cranial lung lobe, the phrenic nerve was isolated. An incision was made in the pericardial sac along the visible pulmonary arterial trunk. The isolation of the trunk from ascending aorta was conducted using angled vascular forceps and Metzenbaum scissors. A Gore-Tex band, approximately 4 mm wide, prepared from a vascular patch (W. L. Gore & Associates, Inc., Newark, NJ, USA), was placed around the trunk (Figure 7). The vessel lumen was gradually reduced by applying sequential titanium vascular clips. The Gore-Tex band was then sutured to the vessel adventitia to prevent it from migrating towards the bifurcation of the pulmonary arterial trunk. The resulting vessel lumen was estimated to be approximately 50% of the original diameter of the pulmonary arterial trunk. The pericardial sac was not sutured. The lung was repositioned, and the chest wall closure was performed. The blood flow velocity in the pulmonary artery (v) was measured using continuous-wave Doppler, yielding a result of 3.8 m/s. This indicates a pressure gradient of 57 mmHg across the banding, i.e., between the right ventricular cavity and peripheral pulmonary arteries (according to the simplified Bernoulli equation: delta ∆P = 4 × v2). This level of increase of the central pulmonary pressure effectively decreases the pressure difference between both ventricles resulting in a decrease in shunting. The echocardiographic parameters before and after PAB are summarized in Table 1. The patient was hospitalized for 3 days after the procedure. The cat’s condition was stable, with a maintained appetite and normal body temperature; dyspnea resolved. A continuous rate infusion of fentanyl and ketamine (Vetaketam, Vet-agro, Lublin, Poland) was continued for the first day, then the dose was tapered by half, followed by switching to oral transmucosal buprenorphine (Bunondol, Polfa Warszawa S.A, Warsaw, Poland) every 8 h. Pain management also included the administration of meloxicam (Metacam, Boehringer Ingelheim Vetmedica GmbH, Ingelheim/Rhein, Germany) at a dose of 0.05 mg/kg subcutaneously (SC) and gabapentin (Neurontin, Pfizer, Sanwich, UK) at a dose of 15 mg/kg per os (PO). Sildenafil was continued at a dose of 2 mg/kg 3 times per day. On the second day post-surgery, the patient required abdominocentesis—400 mL of hemorrhagic fluid was evacuated. The patient was discharged from the hospital on the third day after the surgery. An echocardiographic follow-up 3 weeks post-operation revealed stenosis of the pulmonary trunk (Figure 8 and Figure 9) with an unchanged blood flow velocity of 3.8 m/s (Figure 10), as in the postoperative control immediately after the surgery, with a significant reduction in the size of the right atrium and right ventricle and a visible VSD with bidirectional low-velocity flow (Figure 11 and Figure 12). As of the manuscript publication, the patient is thirteen months post-surgery. No pharmacotherapy is necessary. Fluid does not accumulate in body cavities. The cat has a normal appetite and exhibits behavior typical of a healthy individual—it runs and jumps. In four follow-up echocardiographic studies conducted on the day of surgery, 3 weeks, 2 months, and 9 months after surgery, the results were consistent with those obtained immediately after the procedure. Specifically, the blood flow through the pulmonary artery remained at 3.5–3.8 m/s, and there was a noticeable reduction in the size of the right atrium and right ventricle, as well as a decrease in the defect in the muscular part. The cat has remained in stable condition without the need for pharmacotherapy for 12 months post-surgery and is still alive at the time of reporting.

## 3. Discussion

PAB is a surgical technique employed to treat certain congenital heart defects. In human medicine, it serves mainly as an initial surgical procedure before routine definitive repair, such as an AVC, or as a palliative procedure in extremely severe cases [18,19,20]. The primary indication for PAB is to limit pulmonary blood flow in scenarios of excessive pulmonary circulation caused by significant left-to-right shunts [20]. The physiological objective of this procedure is to protect the pulmonary vasculature by reducing excessive blood flow through the lungs and decreasing the pressure load on the pulmonary precapillaries and capillaries’ veins. This helps prevent pathological remodeling of the pulmonary vessels and the development of PH [19]. A significant risk of PAB is associated with overtightening the band, which can increase the pressure in the right heart so much that the direction of the shunt flow reverses. Therefore, a crucial aspect of PAB is determining the appropriate tightness of the band [21]. It is important to note that if the surgery is performed on growing individuals, an initially appropriate pulmonary artery banding may become inadequate over time as the body grows and oxygen demand increases [22]. This can lead to the development of collateral circulation as the body attempts to compensate for the increased demand for oxygen. Our patient has maintained a constant body weight since the operation. Guidelines suggest reducing the shunt flow velocity to approximately 3.5 m/s in children with AVC defects through the pulmonary artery, measured intraoperatively using epicardial or transthoracic echocardiography. For growing children and animals, it is advisable to consider using expandable bands that can be adjusted via minimally invasive pulmonary artery balloon dilation procedures [21]. Due to the very high costs of complete surgical correction of AVCs, these surgeries are extremely rarely performed in veterinary medicine. Single cases of such procedures have been described in the literature concerning dogs [1]. In some cases, death occurred shortly after surgery due to hypotension [23]. There are also reports of two dogs undergoing surgery involving a patch closure of the interatrial septal defect and plasty of the mitral valve leaflets—one dog remained asymptomatic for 3.5 years after surgery. At the same time, the other showed mild exercise intolerance 15 months post-operation [24]. Reports also exist of commissuroplasty (correction of abnormally fused mitral and tricuspid valve leaflets) and closure with mattress sutures of an interatrial septal defect in a 7-month-old Shiba Inu. The dog remained asymptomatic one year after the procedure [1]. In 2018, a partial atrioventricular canal defect was also described in a Japanese Spitz. The procedure involved a patch closure of the interatrial septal defect and mitral valve plasty, similar to the previous cases. The patient remained asymptomatic for 5 years post-operation [7]. In cats, PAB has been described to reduce right-to-left flow through the shunt in animals with VSDs [19,25,26]. PAB was also performed on a 9-month-old cat with an AVC—the cat showed significant improvement after surgery. In this case, a sterile umbilical tape was used to constrict the pulmonary artery. After placing the band, a flow rate of 2.6 m/s through the main pulmonary artery was achieved and echocardiographic parameters remained unchanged four months after the operation [12]. However, this cat remained on spironolactone and sildenafil therapy, while our patient does not need pharmacotherapy.

In addition to ensuring the appropriate tightness of the band, it is essential to consider potential complications associated with PAB. These may include stenosis or deformation of one or both pulmonary artery branches, impairment of pulmonary valve function, band erosion into the pulmonary artery, development of a pulmonary artery pseudoaneurysm, or localized infection.

However, in fully grown animals diagnosed with an AVC defect—particularly those in poor clinical condition with dyspnea or ascites—PAB can be the treatment of choice, potentially leading to long-term survival with a high quality of life. Further studies are needed to evaluate the long-term outcomes of this procedure in feline patients and define precise criteria for the selection of patients in which the procedure is most likely to be beneficial.

## 4. Conclusions

Pulmonary artery banding (PAB) remains a viable palliative surgical option for managing excessive pulmonary blood flow in patients with atrioventricular canal (AVC) defects. In this case, the procedure successfully stabilized the patient’s condition. Unlike previous reports, where pharmacological support with sildenafil and spironolactone was required postoperatively, our patient has remained stable without needing pharmacotherapy. Thirteen months after the surgery, the cat continues to exhibit normal physiological parameters and no clinical signs of cardiac decompensation, demonstrating the potential benefits of PAB in selected cases.

## Figures and Tables

**Table 1:** Echocardiographic parameters before and after PAB. Left atrial diameter (LAD), aortic diameter (AoD), left atrium-to-aortic ratio (LAD/AoD), right ventricular internal diameter in diastole (RVIDd), left ventricular internal diameter in diastole (LVIDd), left ventricular internal diameter in systole (LVIDs), left ventricular posterior wall thickness in diastole (LVPWd), left ventricular posterior wall thickness in systole (LVPWs), interventricular septal thickness in diastole (IVSd), interventricular septal thickness in systole (IVSs), left ventricular ejection fraction (LVEF), fractional shortening (FS), left ventricular outflow tract maximum velocity (LVOT Vmax), left ventricular outflow tract maximum pressure gradient (LVOT PGmax), right ventricular outflow tract maximum velocity (RVOT Vmax), right ventricular outflow tract maximum pressure gradient (RVOT PGmax), tricuspid annular plane systolic excursion (TAPSE), mitral annular plane systolic excursion (MAPSE), and pulmonary-to-systemic flow ratio (Qp:Qs).

Echocardiographic Measurement	Units	Preoperative	Postoperative
LAD	cm	0.85	0.97
AoD	cm	1.26	1.29
LAD/AoD	1/1	1.48	1.32
RVIDd	cm	1.08	0.30
LVIDd	cm	0.50	0.48
LVIDs	cm	2.15	2.14
LVPWd	cm	0.64	0.40
LVPWs	cm	0.57	0.56
IVSd	cm	1.40	1.20
IVSs	cm	0.64	0.60
LVEF	%	67	77
FS	%	35	43
LVOT Vmax	m/s	1.08	1.04
LVOT PGmax	mmHg	4.70	4.38
RVOT Vmax	m/s	1.01	3.81
RVOT PGmax	mmHg	4.14	58.07
TAPSE	mm	87	43
MAPSE	mm	93	56
Qp:Qs	-	2.89	0.97

## Data Availability

Data are available on request from the corresponding author.
